# Glucagon-like peptide 1 receptor agonists and thyroid cancer: is it the time to be concerned?

**DOI:** 10.1530/EC-23-0257

**Published:** 2023-09-27

**Authors:** Giuseppe Lisco, Anna De Tullio, Olga Disoteo, Giuseppina Piazzolla, Edoardo Guastamacchia, Carlo Sabbà, Vincenzo De Geronimo, Enrico Papini, Vincenzo Triggiani

**Affiliations:** 1 Interdisciplinary Department of Medicine, Section of Internal Medicine, Geriatrics, Endocrinology and Rare Diseases, School of Medicine, University of Bari “Aldo Moro”, Piazza Giulio Cesare, Bari, Italy; 2 Diabetology Unit, ASST Grande Ospedale Metropolitano Niguarda, Milan, Italy; 3 Unit of Endocrinology, Policlinico Morgagni CCD, Catania, Italy; 4 Department of Endocrinology and Metabolism, Regina Apostolorum Hospital, Rome, Italy

**Keywords:** dipeptidyl peptidase IV, glucagon-like peptide 1 receptor agonist, incretin system, medullary thyroid cancer, papillary thyroid cancer

## Abstract

Glucagon-like peptide 1 receptor agonists (GLP-1RAs) have changed considerably the management of type 2 diabetes (T2D). However, recently published data from retrospective cohort studies suggest that chronic exposure to GLP-1RAs in T2D may increase the risk of papillary and medullary thyroid cancer. In this perspective, the role of the incretin system in thyroid carcinogenesis has been reviewed and critically commented on, aiming to understand if the time has arrived to be concerned about the risk. Although evidence suggested, speculative hypotheses should be verified, and further studies are urgently needed to clarify the issue.

## Background

Glucagon-like peptide 1 receptor agonists (GLP-1RAs) have relevant antihyperglycemic effects and provide significant improvement on diabetes-related outcomes such as overweight and obesity, cardiovascular and renal impairment, and nonalcoholic fatty liver disease in randomized clinical trials (1, 2) and real-life evidence (3, 4). Since the approval, a rising interest has orbited around the GLP-1RAs, resulting in a high prescription rate of this class of medications in patients with T2D (and obesity).

Gastrointestinal signs and symptoms are usually the leading adverse events that may occur during the treatment. These encompass nausea, abdominal pain or discomfort, vomiting, stypsis, or diarrhea. Less frequently, pancreatitis and biliary cholic may occur, making it necessary for the GLP-1RA to be discontinued (5). The onset, evolution, and severity of adverse events largely depend on individual susceptibility, the kind of drugs used, titration, and gradual dose escalation. Symptoms are usually mild to moderate and may lessen or disappear over time spontaneously. Severe or persisting symptoms require the GLP-1RA to be discontinued, and an appropriate diagnostic workup is essential to define the etiological diagnosis. After ruling out relevant diseases, the GLP-1RA could be introduced again at the lower tolerated therapeutic dose, with gradual dose escalation. Another drug of the same class could be considered to replace GLP-1RA, which is poorly tolerated. When the previous strategies fail, could not be applied or in case of recurring adverse events, a switch to alternative therapies should be considered. Dietary education is also compulsory to prevent or mitigate the frequency and severity of GLP-1RA-related adverse events. Adequate hydration and fiber consumption, cessation of alcoholic beverage consumption, and eating lighter and smaller meals can be useful advice (6).

The association between chronic exposure to GLP-1RAs and the new onset of thyroid cancer is a growing topic. Cancerogenic studies reported a higher incidence of parafollicular C-cell thyroid hyperplasia and tumors in rodents exposed to serum levels of liraglutide comparable to the therapeutic effect in humans (7). No events of medullary thyroid cancer were described, but a dose eight times higher than the greatest approved dose of liraglutide in humans was related to a potential carcinogenic factor (8). Evidence suggests that GLP-1RAs exert a dose-dependent effect on cell proliferation in thyroid dysplastic or premalignant lesions.

The GLP-1 receptor is a seven-domain G-coupled transmembrane receptor widely expressed on parafollicular C-cells membrane (9). Native GLP-1 induces cell proliferation after binding to the receptor in experimental conditions. It also stimulates the synthesis and secretion of calcitonin in a cAMP-dependent manner. Therefore, GLP-1 may promote rodent thyroid carcinogenesis (10), but insufficient evidence indicates the potential cancerogenic effect in humans (11, 12). Nevertheless, the possible cause–effect and dose-dependent relationships between chronic exposure to GLP-1RAs and the risk of thyroid cancer in T2D and obese individuals have not been ruled out.

## Approach to the matter

Since 2012, data from the postmarketing surveillance have revealed an increase in the incidence of thyroid carcinomas in patients taking exenatide, thus highlighting a safety issue for the GLP-1RA. The specific concern was to understand better if the increase in the incident discovery of thyroid carcinomas could have been only a concomitant phenomenon (e.g. increased surveillance of thyroid nodules), or instead, the GLP-1RAs per se could have stimulated the proliferation of intra-thyroid preneoplastic lesions by acting as promoting factors (13, 14, 15, 16).

Since then, regulatory agencies have recommended a careful and comprehensive anamnestic and thyroid imaging collection in candidate patients to receive GLP-1RAs before treatment initiation and through the follow-up to evaluate long-term effects (17, 18, 19).

## The incretin system and thyroid cancer

A pivotal study found that the dipeptidyl peptidase type IV (DPP-IV), also known as cluster differentiation 26 (CD26), was expressed widely on the cell surface of follicular thyroid carcinoma samples that were clinically associated with distant metastasis (20). Takana *et al.* discovered that the frequency of CD26-positive cells, the level of activity staining, and the degree of CD26 messenger ribonucleic acid expression were significantly higher in 57 thyroid samples (pathology: 55 papillary and five follicular carcinomas) than in 58 controls (Graves’ disease) (21). Kholová *et al.* found that positive immunostaining for DPP-IV over 50% of positive cells on 254 thyroid specimens with confirmed histology had an overall diagnostic accuracy of 93% to differentiate follicular from oncocytic tumors, and in distinguishing between nuclear atypia in colloid goiter with regressive changes and cystic papillary carcinoma (22). These data suggested that DPP-IV could be a potential marker of thyroid malignancy and prognosis in patients diagnosed with thyroid carcinoma or indeterminate cytological findings.

Despite the role of DPP-IV as a diagnostic marker of thyroid malignancy, it is unclear whether the enzyme could also be a potential therapeutic target in differentiated thyroid cancer. A mechanistic study found that saxagliptin, a DPP-IV inhibitor approved for T2D, increased the nuclear and cytoplasmic accumulation of the nuclear factor erythroid 2-related factor 2 (NRF2), which is a transcription factor involved in the regulation of oxidative stress, cell migration, and invasion. More precisely, the saxagliptin-induced overexpression of NRF2 increased the synthesis of heme oxygenase 1, matrix metalloprotease 2, and vascular endothelial growth factor providing the human thyroid carcinoma cells migratory and invasive capabilities (23). Nevertheless, other results suggested that DPP-IV silencing or inhibition with sitagliptin constrained cell proliferation and the epithelial-to-mesenchymal transition in papillary thyroid cancer cells via suppression of the mitogen-activated protein kinase pathway (24), and the growth factor-β receptor I expression (25, 26). The clinical relevance of the mentioned findings is inconclusive. The results of a systematic review and meta-analysis of randomized and observational studies did not find any significant association between the use of DPP-IV inhibitors and the risk of thyroid cancer (27). To conclude, available data suggested neither protective nor detrimental effects of DPP-IV inhibitors in T2D. The administration of DPP-IV inhibitors as a potential therapeutic strategy to reduce the risk of thyroid cancer in T2D patients or to prevent disease progression in high-risk patients could be an issue.

Evidence from rodent studies suggested that GLP-1RAs may stimulate parafollicular C-cells proliferation, potentially affecting the risk of new-onset medullary thyroid neoplasms. Observation studies in humans did not confirm the potential cancerogenic effect of GLP-1RAs, such as Liraglutide or Exenatide, in humans (8, 28). The determining factor of this difference could be attributable to the level of tissue GLP-1 receptor expression across the species. GLP-1 receptors are expressed in a percentage not exceeding 30% of human thyrocytes and parafollicular C-cells (29), and they were also found in a lower rate of papillary thyroid carcinomas (30).

Therefore, the role of GLP-1 receptors as a diagnostic and prognostic marker of medullary and differentiated thyroid cancers is unclear, and the relationship between chronic exposure to GLP-1RAs and cell proliferation in this site could be well elucidated. Dulaglutide, a once-weekly long-acting GLP-1RA, did not affect serum calcitonin levels during a 6-month follow-up of 56-year-old women with preexisting elevation of serum calcitonin and medullary thyroid carcinoma. The patient was recruited for the dose-finding trial AWARD-5 and received dulaglutide 2 mg weekly for 6 months (31). Immunostaining found that GLP-1 receptors were not expressed on the C-cell surface in histologic thyroid samples, thus explaining why the calcitonin level, the biomarker of C-cell proliferation, was unaffected over the follow-up. While in rodents GLP-1RAs stimulate the calcitonin secretion in an adenosine monophosphate cyclic (cAMP)-dependent manner, in humans, the hormone secretion is independent of the GLP-1 agonism and only very high cytoplasmatic cAMP concentration serves calcitonin secretion (32). As GLP-1 receptors are expressed in a minority of follicular and parafollicular C-cells in thyroid cancer and healthy thyroid samples, the cancerogenic role of GLP-1RAs must be better elucidated. Case series reported from clinical studies did not reveal the detection of medullary thyroid cancer in humans taking liraglutide, and the same was for sequential changes in calcitonin levels in thousands (33). Data from the cardiovascular outcome trials suggested that 3-year exposure to GLP-1RAs did not affect the calcitonin level nor increase the risk of new diagnoses of medullary thyroid carcinomas (34, 35, 36, 37). Only a few adjudicated events of thyroid malignancy were reported with both subcutaneously injectable and oral semaglutide without any relevant change in serum calcitonin level over the follow-up (38). Overall, these data contrast the results of a retrospective analysis of spontaneous reports during the postmarketing pharmacovigilance form the EudraVigilance database. Indeed, over 11,000 thyroid cancer diagnoses from more than 6.6 million people were recorded in the database, and 236 cases (64 medullary thyroid cancers) were reported among GLP-1RAs users. Thyroid cancer alerts were generated more frequently in T2D patients on exenatide, liraglutide, and dulaglutide than other mediations (39).

## Real-world data

A recently published French study (40) provided the scientific community with helpful information. Around 48,000 people (47,746) with established diagnoses of T2D and who were on ‘second-line’ treatment between 2006 and 2018 were included in the retrospective cohort. The cases of thyroid cancer were identified after the consultation of hospital discharge diagnoses and medical/surgical procedures from 2014 to 2018. The exposure to GLP-1RAs was calculated over 6 years preceding a 6-month delay between the first GLP-1RA prescription and the evidence of a thyroid event. Exposure was also graded as brief (≤1 year), intermediate (1–3 years), and prolonged (over 3 years). Cases were coupled according to a risk-based sampling up to 20 years of age and gender-matched controls with similar diabetes evolution. The estimation of thyroid cancer risk due to GLP-1RA exposure was calculated by an adjusted logistic regression method (goiter, thyroid dysfunction, antihyperglycemic medications other than GLP-1RAs, and a validated index of social poverty estimation). Given the approach, 2,562 patients diagnosed with thyroid carcinoma were matched to 45,184 controls.

The exposure to a GLP-1RA from 1 to 3 years was associated with a statistically significant increase in the risk of all thyroid carcinomas by 58% (hazard ratio or HR: 1.58; 95% interval confidence or 95% IC: 1.27–1.95), and medullary thyroid cancer by 78% (HR: 1.78; 95% IC: 1.04–3.05). Patients included in the other two groups of exposure (≤1 year and over 3 years) also showed a higher risk of incident thyroid malignancy, although the intergroup difference was not statistically relevant due to a low number of cases (40).

## Discussion

A potential association between chronic exposure to GLP-1RAs, 1–3 years before, and increased risk of new-onset thyroid carcinoma has been provided. The study further highlights the issue beyond previously published data from real-world evidence on the oncology safety of GLP-1RAs (41). However, data should be read cautiously (42).

First, the risk and benefits should always be addressed and balanced comprehensively before providing a conclusive judgment. GLP-1RAs, especially long-acting ones, have been demonstrated to significantly reduce the number of composite adverse cardiovascular events and improve composite renal outcomes (37, 43, 44, 45, 46). In terms of the absolute number of events, cardiovascular and renal benefits are expected to significantly overwhelm the risk of onset, recurring, or advanced thyroid carcinomas (47, 48).

Second, the cohort included patients on ‘second-line’ treatment, such as oral gliptins, thiazolidinediones, sulfonylureas, glinides, GLP-1RAs, and basal insulin. Patients on GLP-1RAs would have assumed the medication at least 6 months before diagnosing a thyroid event (carcinoma) for considering a cause–effect relationship. The absence of an adequate wash-out period does not allow for the precise identification of new GLP-1RAs users, increasing the risk of bias in risk estimation studies. Furthermore, the active comparator rather than the placebo is an additional source of bias, as it is unclear if other drugs over GLP-1RAs may have protective, neutral, or detrimental effects on thyroid carcinogenesis.

Third, the incidence of thyroid cancer ranks ninth worldwide. Although it is more frequently diagnosed in women than men, in absolute terms, thyroid cancer is not a common malignancy, despite the increase in the number of new diagnoses over the last three decades. Moreover, thyroid carcinomas are under the 20th place for mortality, as the rise in the number of new diagnoses could probably be attributable to overdiagnosis of thyroid nodules and carcinomas (such as differentiated papillary microcarcinomas with an almost always indolent prognosis) rather than an absolute increase of absolute thyroid carcinoma incidence. Overdiagnosis and overtreatment of thyroid nodules are generally not recommended, as they appear not to be cost-effective procedures and generate confusing evidence without affecting the natural history of the disease (49, 50, 51). T2D is a risk factor for thyroid cancer, and the use of GLP-1RAs has prompted clinicians to provide more frequent thyroid ultrasound monitoring before and after prescribing this class of drugs. Especially in a real-life setting, the number of thyroid neoplasms diagnoses may be overestimated. To confirm this suggestion, the incidence of medullary carcinoma, one of the most aggressive forms among thyroid carcinomas, was above 15% in the French study and was exceptionally higher compared to what is described in the general population (<2%).

As another limitation, other variables, also known as thyroid cancer risk factors, could have been included in the logistic regression model, such as family history and obesity. Obesity is closely related to T2D from an epidemiological and pathophysiological point of view (52). It is known that obesity is associated with around 30% increased risk of differentiated, but not medullary, thyroid carcinoma (53), with metrics usually used to assess body weight excess and adipose tissue overload being linearly associated with a greater risk of thyroid cancer (54). The risk seems to be affected by gender, with women being exposed to a greater risk of differentiated thyroid cancer than men are, although a healthy body weight reduces the risk in both genders (55, 56). Moreover, obesity is significantly associated with several adverse histological characteristics of papillary thyroid cancer at diagnosis, including larger tumor size, multifocality, extrathyroidal extension, lymph nodal involvement, and BRAF mutation (57, 58, 59). Several studies suggested that the leading mechanisms explaining how weight excess can affect the risk of differentiated, mostly papillary, thyroid cancer are in the pro-inflammatory background (interleukin 6 and tumor necrosis factor alpha), insulin resistance, hyperglycemia, dyslipidemia, and altered signaling of thyroid-stimulating hormone, insulin growth factor 1, leptin, adiponectin, and androgens, ultimately promoting thyroid cell proliferation (60, 61, 62, 63). Given the evidence, more attention should be placed on obesity as it typically characterizes the phenotype of T2D patients who are candidates to receive a GLP-1RA.

Last, 6 months of exposure to GLP-1RAs as the shortest time limit to consider a potential cause–effect relationship may be no longer enough to produce clinically relevant effects on carcinogenesis, potentially affecting an increase in the number of judged events of thyroid carcinomas not actually due to the beginning and short-term exposure to a GLP-1RA. However, a few percent of premalignant lesions and malignant thyroid carcinomas express GLP-1 receptors, thus being exposed to the proliferative effect of GLP-1RAs. The proliferation-promoting effects of GLP-1RAs could therefore be suspected in the case of patients with occult premalignant lesions or malignant thyroid nodules who are on the medication. Although the hypothesis is purely speculative, it could be intriguing to verify it by stratifying the analyses and reassessing the risks based on the presence (or absence) of GLP-1 receptors on thyroid samples.

## Conclusion

Further studies are urgently needed to provide more evidence on the role played by the incretin system in thyroid carcinogenesis. It is reasonable to continue prescribing GLP-1RAs due to the evidence of more relevant and well-recognized antihyperglycemic, cardiovascular, and renal benefits, with the management staying within current recommendations.

## Declaration of interest

The authors declare that they have no conflict of interest.

## Funding

None to declare.

## Author contribution statement

GL and VDG conceived the perspective; GL, VDG, VT, and ADT searched databases and selected appropriate references; GL and ADT drafted the manuscript; VGD, VT, OD, and GP read the text and gave feedback; VT, EP, CS supervised the draft and approved the final version of the manuscript. All authors approved the manuscript submission.
